# Old concepts, new challenges: adapting landscape-scale conservation to the twenty-first century

**DOI:** 10.1007/s10531-016-1257-9

**Published:** 2016-12-05

**Authors:** Lynda Donaldson, Robert J. Wilson, Ilya M. D. Maclean

**Affiliations:** 1 Environment and Sustainability Institute, University of Exeter, Penryn Campus, Cornwall, TR10 9FE UK; 2 College of Life and Environmental Sciences, University of Exeter, Exeter, EX4 4PS UK

**Keywords:** Habitat degradation, Habitat network, Island biogeography, Landscape ecology, Metapopulation, Conservation strategies, Climate change

## Abstract

Landscape-scale approaches to conservation stem largely from the classic ideas of reserve design: encouraging bigger and more sites, enhancing connectivity among sites, and improving habitat quality. Trade-offs are imposed between these four strategies by the limited resources and opportunities available for conservation programmes, including the establishment and management of protected areas, and wildlife-friendly farming and forestry. Although debate regarding trade-offs between the size, number, connectivity and quality of protected areas was prevalent in the 1970–1990s, the implications of the same trade-offs for ongoing conservation responses to threats from accelerating environmental change have rarely been addressed. Here, we reassess the implications of reserve design theory for landscape-scale conservation, and present a blueprint to help practitioners to prioritise among the four strategies. We consider the new perspectives placed on landscape-scale conservation programmes by twenty-first century pressures including climate change, invasive species and the need to marry food security with biodiversity conservation. A framework of the situations under which available theory and evidence recommend that each of the four strategies be prioritized is provided, seeking to increase the clarity required for urgent conservation decision-making.

## Introduction

After failing to meet the 2010 Convention on Biological Biodiversity (CBD) targets (Butchart et al. 2010), the global community has been offered a second chance to halt biodiversity declines by 2020 through the CBD’s Aichi targets (CBD 2011). Current financial resources available to meet these targets are insufficient (McCarthy et al. 2012) and in consequence there is urgent need for conservation planners and practioners to have sufficient information to select and employ efficient, cost-effective actions (Williams et al. 2005). Nevertheless, there is much debate regarding the most effective means for adapting conservation to accelerating environmental change (Hodgson et al. 2009c), leading to an extensive literature that presents some apparently conflicting messages to those involved in conservation planning and decision-making.

**Fig. 1:**
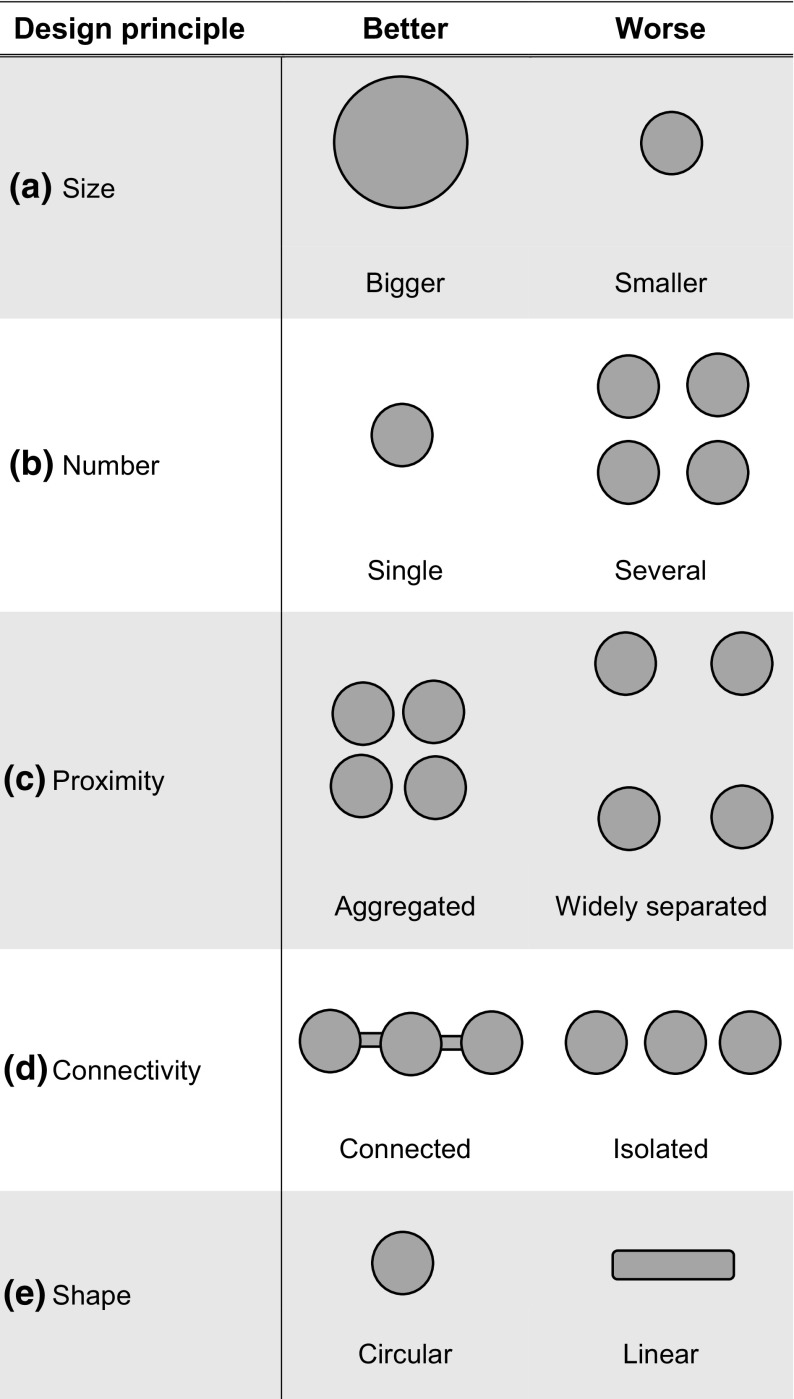
Suggested geometric principles for nature reserve design derived from Diamond (1975). In all cases, species extinction rate would be expected to be lower on the *left* (*better*) than on the *right* (*worse*)

Classical approaches to increase the effectiveness of protected area designation and management have drawn upon the theories of island biogeography (MacArthur and Wilson 1967) and metapopulation dynamics (Levins 1969; Hanski and Gilpin 1991; Hanski 1999). In these approaches, the four main trade-offs among the size, number, quality and connectivity of protected areas can be summarised by Diamond’s (1975) outline of geometric principles for the design of nature reserves (Fig. 1). Since the 1990s, however, conservation actions have evolved from a primarily reserve-based approach to give greater consideration to landscape-scale processes (Opdam and Wascher 2004; Watts et al. 2010), partly because climate change and increased habitat fragmentation have led to increasingly dynamic patterns of colonization and extinction (Heller and Zavaleta 2009). Landscape-level conservation initiatives include the Pan-European Ecological Network (Jongman et al. 2011) and “greenways” in the USA (Ahern 2004). In England, the recent “*Making Space for Nature*” report (Lawton et al. 2010) summarized the recommendations of a now substantial scientific literature to increase the effectiveness of protected area networks in fragmented landscapes in four simple words: “more, bigger, better and joined”. The report recommended, in a priority hierarchy: (1) improving the quality of habitat, (2) increasing the size and (3) number of sites, and (4) enhancing connectivity among sites for conservation. In the UK, these recommendations are incorporated into biodiversity policy (Department for Environment, Food and Rural Affairs (DEFRA) 2011) and increasingly inform planning and management by conservation agencies and organisations working to maintain and restore habitats in the UK’s highly fragmented landscapes.

The Lawton et al. (2010) report provides valuable recommendations regarding the UK’s network of protected sites, but global variation in land-use history, levels of fragmentation and biogeographic context drive a need to determine more widely for conservation practitioners the circumstances under which increasing the area, number, connectivity and quality of conservation sites is most effective. Published research seldom tackles trade-offs among all 4 approaches together to assist with the transition from theory to practical application (but see Hodgson et al. 2011a). Moreover, since the origin of the principles of reserve design, the challenges faced by biodiversity have evolved from emphasis on land use change in the twentieth century (Sala et al. 2000), to include a rapid rise in impact from climate change, invasive species and pollution, alongside continuing pressures from overexploitation in the twenty-first century (Millennium Ecosystem Assessment 2005; Urban 2015). The ability of the natural environment to provide ecosystem services is declining as a result of increasingly degraded habitats (Millennium Ecosystem Assessment 2005) which, coupled with increasing human populations, impacts the ability to marry food security with conservation. These pressing issues necessitate a shift in focus from the simplistic interpretation of what was originally thought to be best; effectively factoring new challenges into decision making from a reemphasis of original ideas, to modifying classical theory to adapt to a world of accelerating environmental change. To our knowledge, research to date has not addressed these challenges alongside their impact on assumptions from classical reserve design.

Here, we synthesize concepts associated with landscape-scale approaches to conservation, and offer a practical blueprint for effective decision making, highlighting how our priorities change in the context of twenty-first century challenges including climate change, the spread of invasive species and food security, which were largely unforeseen when the original approaches were devised (Table 1). We present the four axes of reserve design in the order of decreasing importance as proposed by Lawton et al. (2010), but consider trade-offs first associated with habitat quality, then between size and number of reserves, and finally consider the importance of connectivity and how to achieve it. The nexus between conservation theory and modern day application is invariably tangled by complexities and practicalities. We aim to provide conservation decision-makers with the information they need to make informed choices on the most effective action given, and plot a path through some of this tangle.

**Table 1:** Overview of the main considerations and summary of evidence from key supporting references associated with the most effective strategy between better, bigger, more and more-connected sites

Consideration				Recommended strategy	Summary of evidence
Goal	Multiple spp.			*Heterogeneity*	Greater species diversity with habitat variety (Rosenzweig 1995) e.g. (see Benton et al. 2003; Báldi 2008)
				*Bigger*	Species Area Relationship states that larger sites hold more species (MacArthur and Wilson 1967; Diamond 1975) through habitat diversity, area per se, concentration of resources, edge effects (Connor and McCoy 2001) e.g. (Brückmann et al. 2010)
				*More sites*	High rates of immigration (Fahrig 2003) and wide variety of habitat (Dover and Settele 2009; Oliver et al. 2010), supporting a range of species
				*More connected*	Both area and isolation influences the number of species a site can hold (Diamond 1975) e.g. plants (Damschen et al. 2006), butterflies (Brückmann et al. 2010)
	Single sp.	Habitat preference	Interior	*Bigger*	Less edge; higher area: edge ratio (Bender et al. 1998)
			Edge	*Homogeneity*	Availability for colonization (Thomas et al. 2012)
				*More sites*	Edge effects (Bender et al. 1998)
				*More connected*	Corridors provide high edge: area ratio (Haddad 1999; Haddad and Tewksbury 2005)
			Specialist	*Homogeneity*	Specific habitat requirements (Ye et al. 2013) e.g. birds (Root 1998; Devictor et al. 2008), butterflies (Dennis et al. 2013)
				*Bigger*	Matrix habitat not suitable, negatively affected by habitat fragmentation, avoid edge (see Brückmann et al. 2010); e.g. butterflies (Dover and Settele 2009; Brückmann et al. 2010) and plants (Dover and Settele 2009)
				*More connected*	Less likely to occur in matrix than generalists (Brückmann et al. 2010) e.g. butterflies (Haddad 1999; Brückmann et al. 2010; Dennis et al. 2013) and plants (Brückmann et al. 2010)
			Generalist	*Heterogeneity*	Less sensitive to quality (Ye et al. 2013) e.g. birds (Devictor et al. 2008), butterflies (Oliver et al. 2010)
				*More sites*	Occur in matrix, occupy smaller isolated patches (Dennis et al. 2013)
				*Less connected*	More likely to exist in matrix between sites (Lees and Peres 2009; Brückmann et al. 2010)
		Habitat requirements	Migratory	*Heterogeneity*	Buffers variation in resources through time (Benton et al. 2003)
				*More sites*	Move between sites to meet habitat requirements (Bender et al. 1998)
				*More connected*	Move between sites to meet habitat requirements (Benton et al. 2003; Donald and Evans 2006)
		Range size	Large	*Bigger*	Less prone to extinction in larger sites (Di Minin et al. 2013) and less at risk from human-wildlife conflict (Abele and Connor 1979; Woodroffe and Ginsberg 1998)
				*More connected*	Enable movement between sites (Rosenberg et al. 1997; Donald and Evans 2006; Lawton et al. 2010) and increase recolonization rates (Di Minin et al. 2013)
			Small	*Heterogeneity*	More vulnerable to environmental change, buffers these effects (Oliver et al. 2010)
				*More sites*	Smaller sites are sufficient for range requirements (Abele and Connor 1979)
		Body size	Large	*Bigger*	Larger bodied species have larger range sizes (Abele and Connor 1979; Cardillo et al. 2005)
			Small	*More sites*	Smaller range sizes thus smaller sites are sufficient (Abele and Connor 1979; Cardillo et al. 2005)
		Dispersal capability	High	*More sites*	Capacity to move between sites (Nicol and Possingham 2010)
				*Less connected*	Links would have limited worth (Bennett 2003)
			Intermediate	*Bigger*	Lower mortality rate associated with less emigration and failure to locate site (Thomas 2000)
				*More connected*	Assist with locating patches (Thomas 2000), especially matrix restoration (Donald and Evans 2006)
			Very poor/sedentary	*Homogeneity*	Require good quality habitat (Ye et al. 2013)
				*Bigger*	Less need for movement (Öckinger and Smith 2006; Nicol and Possingham 2010)
				*More connected*	Assist with dispersal, providing within dispersal range (Doerr et al. 2011)
		Dispersal mode	Animal-borne	*More connected*	Assists with animal movement (Brudvig et al. 2009)
			Wind-borne	*More sites*	More edge to reach non-target habitat (Brudvig et al. 2009)
				*Less connectivity*	Unaffected by direct connectivity (Brudvig et al. 2009)
		Population viability	High	*More sites*	Metapopulation persistence (higher turnover of local extinction and recolonization) (Drechsler and Wissel 1998)
				*More connected*	Metapopulation persistence (Drechsler and Wissel 1998)
			Low	*Homogeneity*	Higher population growth (Thomas et al. 2001; Griffen and Drake 2008; Ye et al. 2013)
				*Bigger*	Greater population carrying capacity (Griffen and Drake 2008)
Landscape attributes^a^	Fragmented			*Heterogeneity*	Less vulnerable to climate change and extreme events in fragmented landscapes (Opdam and Wascher 2004)
				*More sites*	Species will be more adapted to live in fragments (Schnell et al. 2013)
				*More connected*	Movement between habitats is important (Isaak et al. 2007; Dennis et al. 2013)
	Continuous			*Bigger*	Species are poorly adapted to live in small fragments (Schnell et al. 2013)
Climate variability (risk of disease/environmental disturbance) and vulnerability to climate change^a^	High variability + low vulnerability			*Heterogeneity*	Buffers stochastic extinctions from environmental disturbance (Opdam and Wascher 2004; Hopkins et al. 2007; Piha et al. 2007; Dover and Settele 2009), stabilizes populations (Oliver et al. 2010)
				*More sites*	Spreads risk of extinction (Groeneveld 2005; Dover and Settele 2009; Oliver et al. 2010) and encourages recolonization through “stepping stone” habitat (Schnell et al. 2013)
				*Less connected*	Spreads risk of extinction and reduce impact (Simberloff and Cox 1987; Williams et al. 2005)
	Low variability + high vulnerability			*Homogeneity*	Location for colonization and thus range shift (Hodgson et al. 2011a; Thomas et al. 2012)
				*Bigger*	Larger source populations to facilitate range shift (Hodgson et al. 2009c, 2011a)
				*More sites*	Promote rapid movement through stepping stone habitat (Hodgson et al. 2012; Magris et al. 2014)
				*More connected*	Higher probability of colonization and thus range shift (Heller and Zavaleta 2009; Hodgson et al. 2012; Lawson et al. 2012)
	Low variability + low vulnerability			*Homogeneity*	Strong patch quality-occupancy relationship in static habitat (Hodgson et al. 2009b)
				*More connected*	Strong connectivity-occupancy relationship in static habitat (Hodgson et al. 2009b)
Economics and ownership^a^	Limited funds			*Homogeneity*	Protect currently intact environments, restoring habitat is financially expensive and time consuming (Possingham et al. 2015)
				*Bigger*	Lower unit/area management costs (Simberloff and Abele 1976; Radchuk et al. 2012) since rely on natural processes (Lawton et al. 2010) and require low maintenance (Williams et al. 2005)
				*More connected*	Balance costs associated with SLOSS (Simberloff and Abele 1976); and cost effective to use existing natural connections or man-made structures (Lawton et al. 2010)
	Surrounding land ownership			*More sites*	Enlarging sites not possible (Dover and Settele 2009; Doerr et al. 2011)
				*More connected*	Discourage species use of neighboring habitat (Hartter and Southworth 2009)
	No surrounding land ownership			*Bigger*	Encourage protection of more space for nature (Dover and Settele 2009)

^a^Evidence for the strategy to adopt amid new challenges not conventionally considered

## Quality in a changing world

Enhancing habitat quality has traditionally been a crux of reserve-based conservation (New et al. 1995). Numerous studies demonstrate that improved habitat quality reduces the amount of habitat needed to sustain populations of species (Lawton et al. 2010) and following the shift in focus to reserve configuration and connectivity promoted by metapopulation biology, many others highlight the role of habitat quality in enhancing metapopulation persistence in fragmented landscapes (e.g. Verboom et al. 1991; Thomas 2000; Thomas et al. 2001; Fleishman et al. 2002; Resetarits and Binckley 2013). In the face of climate change, improving habitat quality through better in situ management is now generally regarded as the most important step for biodiversity conservation (Lawton et al. 2010; Hodgson et al. 2011a; Resetarits and Binckley 2013; Greenwood et al. 2016). Enhancing quality can also effectively enhance connectivity by increasing the number of potential dispersers (Hodgson et al. 2009c), and promote the ability of species to shift in response to a warming climate (Hodgson et al. 2009c, 2011a; Lawson et al. 2013). Simply preserving intact habitat, as opposed to enhancing its quality can also be an effective approach when time and money is serverly limited (Possingham et al. 2015).

### Homogeneity or heterogeneity?

Two broad approaches have been suggested as means of enhancing quality: providing more optimal habitat (homogeneity) or increasing heterogeneity, generally achieved through restoration of existing degraded habitat, or managing intact areas. The existing trade-offs between these two approaches have seldom been recognised, yet influence the outcome and overall effectiveness of management. Studies have demonstrated the positive influence of creating more optimal habitat on population size (Thomas et al. 2001; Ye et al. 2013), dispersal success (Ye et al. 2013), and population growth (Griffen and Drake 2008). In turn, providing more optimal habitat can influence extinction and colonization rates (Thomas et al. 2001; Fleishman et al. 2002; Franken and Hik 2004; Lawton et al. 2010; Thomas et al. 2012; Resetarits and Binckley 2013; Ye et al. 2013), providing source populations and habitats for colonization, which enhance the capacity of species to shift with climate change (Thomas et al. 2012). In contrast, greater habitat heterogeneity buffers the effect of environmental fluctuation compared to homogenous habitats, encouraging population stability (Opdam and Wascher 2004). Since the frequency of extreme climate events is likely to increase (IPCC 2007), the buffering effects of habitat heterogeneity could now be important for climate change adaptation (Piha et al. 2007; Maclean et al. 2015). Moreover, irrespective of changes in the frequency of extreme events, the suitability of various habitat types for species is likely to change with climatic change. Thus providing greater habitat variety is viewed as a particularly effective adaptation strategy, over and above homogeneity, within a dynamic environment (Oliver et al. 2010).

Given these contrasting approaches towards in situ management and supporting evidence for each method particularly in the face of environmental change, evidence and understanding of the circumstances under which approach to follow is key. This decision partly depends on whether the primary conservation objective is single species conservation versus the protection of multiple species. Although many conservation programmes and the direct outcomes through which their success is measured tend to be single-species oriented, contingent on funding and/or legislation, an underlying assumption is that these measures will benefit other species or the community as a whole through umbrella or focal species effects (Bennett et al. 2015). The habitat characteristics that signify high quality are likely to be species-specific (Mortelliti et al. 2010) and so for individual species conservation programmes, habitats with high quality resources geared towards the focal species represent the preferred approach. However, this is only true in more stable environments (Johnson 2007), or by ensuring that habitat management itself offsets climatic changes (Greenwood et al. 2016). If the stated goal is to conserve multiple species, enhancing heterogeneity, and thus habitat variety, is likely to be more effective (Oliver et al. 2010). Field mosaics, for example, have been shown to benefit various species of birds and invertebrates, and the loss of heterogeneity through agricultural intensification is one of the reasons for biodiversity declines on farmland (see Benton et al. 2003). Amid the modern-day landscape, however, an increase in habitat variety can also lead to an increase in species richness of invasive species (Pyšek et al. 2002) which can result in undesirable effects on the community structure of native species (Levine et al. 2003). It should also be recognised that optimal quality can promote range shifts for other (non-focal) species and thus could still form part of multiple species conservation in the face of climate change (Lawson et al. 2013), and can be a benefical approach even if a particular focal species is replaced by non-target species as ranges move (Hodgson et al. 2009c).

Alternatively, if concentrating efforts on a single species, the requirements of that species and location within its geographic range are important. Specialist species are often more threatened than generalist species, more sensitive to within-patch variation in quality, and thus benefit from more homogeneous environments (Devictor et al. 2008; Ye et al. 2013). Nevertheless, if specialist species also have small geographic ranges and restricted populations, they are more vulnerable to environmental change (e.g. Davey et al. 2012) and could benefit from the buffering effects of habitat heterogeneity (Oliver et al. 2010), as has been shown to be the case for birds (e.g. Root 1998) and species of British butterflies (Dennis et al. 2013). For those with different habitat requirements at varying stages of their lifecycle, habitat variability may be beneficial or essential (Johnson 2007; Oliver et al. 2010); though in this context, heterogeneity can be considered a component of optimal habitat quality.

In terms of location, the position of a species in its range and within the landscape influences levels of exposure to temporal fluctuations in conditions and resource availability that can be buffered by spatial heterogeneity (Opdam and Wascher 2004; Dover and Settele 2009). Populations at the edge of species’ ranges or in anthropogenically fragmented landscapes typically occupy smaller and more isolated areas of habitat. If dispersal between populations and rescue/recolonization are inhibited (Opdam and Wascher 2004), then promoting persistence through enhanced local habitat heterogeneity may be particularly pertinent (Lawton et al. 2010). Nevertheless, prevailing conditions at the edges of species’ geographic ranges are expected to represent the environmental limits at which populations can persist, so ensuring some optimal areas of habitat are present both in reserves (Thomas et al. 2001) and at the edge of the species range to allow for recolonization (Thomas et al. 2012), is essential. Finally, for species residing in fragmented landscapes consisting of networks of smaller patches (Moilanen and Hanski 1998), the effects of habitat quality on colonization and extinction may be less important than area and isolation. In this case, the creation of bigger, more connected sites will be more effective than simply improving patch quality.

## Space for nature

Traditionally the theories of island biogeography (MacArthur and Wilson 1967; Simberloff and Abele 1976) and metapopulation dynamics (Moilanen and Hanski 1998; Hanski 1999) emphasize the role of habitat area in influencing local population viability, and have contributed to the prioritization of larger reserves over smaller ones in conservation planning (Williams et al. 2005; Lawton et al. 2010). Nevertheless, there is conflicting evidence suggesting that several small reserves may be more effective than a single large one of equivalent total area (see Ovaskainen 2002). The “SLOSS” (Single Large or Several Small) debate between these two perspectives originated in the 1970s and remains contentious despite numerous attempts at resolution (Tjørve 2010). In the current context of challenges now faced by biodiversity, each strategy continues to offer different pros and cons depending on the challenge in question.

Larger sites have classically been favoured for their greater carrying capacities (Hanski 1999) and consequently, are less vulnerable to extinction from environmental and demographic stochasticity (Diamond 1975; Huxel and Hastings 1999; Franken and Hik 2004; Griffen and Drake 2008). Since climate change is coupled with an increase in extreme weather events (IPCC 2007), buffering the impact of this with larger population sizes is an effective strategy. In the ringlet butterfly (*Aphantopus hyperantus*), for example, larger sites were less sensitive to droughts and promoted faster population recovery (Oliver et al. 2013). Larger sites also offer a reduced risk of inbreeding (Groeneveld 2005) and loss of genetic variability due to drift (Jarvinen 1982), potentially increasing intrinsic adaptability to environmental change (see Merilä 2012). The main appeal for larger sites within modern-day landscape-scale conservation, however, is the capacity to enhance range shift. Large source populations in reserves enhance colonization of surrounding habitat, supporting metapopulation persistence in highly fragmented landscapes (Wilson et al. 2002; Lawson et al. 2012), thus facilitate range shifts in the face of climate change (Hodgson et al. 2011b). Moreover, large sites have been advocated for their ability to support greater species richness (e.g. Connor and McCoy 2001; Lees and Peres 2006; Hartter and Southworth 2009; Lawton et al. 2010; Dennis et al. 2013) and may enhance the capacity of natural areas to provide ecosystem services such as pollination (Kremen et al. 2004; Palmer et al. 2004; Klein et al. 2007).

Nevertheless, contrary to classical theory, creating bigger sites is not consistently effective when accounting for modern-day challenges to biodiversity. Landscapes are becoming increasingly threatened with large correlated environmental disturbances (Huxel and Hastings 1999) and exposed to frequent disease epidemics (Jarvinen 1982), under which the presence of a large continuous block of habitat can increase extinction risk and reduce the chance of recolonization from surrounding populations (Groeneveld 2005; Schnell et al. 2013). Whilst large protected area size can reduce propagule pressure from invasive species because of a reduced perimeter:area ratio (Hulme et al. 2014), effective monitoring and control of invasive species can be more difficult to achieve in larger protected areas (Foxcroft et al. 2013). There are also social and cultural constraints to the designation of protected areas that were not considered by original solutions to the SLOSS debate (Williams et al. 2005), such that increasing habitat area for conservation is often not possible within modern landscapes (Doerr et al. 2011).

In contrast, immigration rates to multiple smaller conservation sites can often be higher (Fahrig 2003), the landscape-scale risk of extinction lower (Hartley and Kunin 2003; Groeneveld 2005; Nicol and Possingham 2010) and the variety of habitat greater (Dover and Settele 2009; Oliver et al. 2010). Consequently, landscapes with several smaller sites can hold more species than a single large site (Simberloff and Abele 1976; Groeneveld 2005; Báldi 2008; Rybicki and Hanski 2013), but could be missing habitat specialist or interior species with large body size (Cardillo et al. 2005) or resource and area requirements (e.g. Oertli et al. 2002; Ye et al. 2013). Whether or not conservation managers are directly focusing on single or multiple species, recognition of the dynamic responses of populations and metapopulations to environmental change calls for the siting of reserves to support the persistence of species rather than simply the representation of as many as possible (see Margules and Pressey 2000; Kukkala and Moilanen 2013). Planning tools have been developed to examine how the area and configuration of reserves can optimise both persistence and the complementarity of species protected (e.g. Moilanen et al. 2005).

In terms of specific implications of accelerating environmental change for the SLOSS debate, studies frequently fail to specify the extent to which invasive species contribute to the increased richness of landscapes with multiple smaller sites (Pyšek et al. 2002). However, providing an increased number of so-called “stepping stone” habitats or protected areas can enhance the speed of colonization of new landscapes, increasing the ability of species to track climate change (Hodgson et al. 2012), both in terrestrial (Lawson et al. 2012) and potentially marine environments (Magris et al. 2014).

### Bigger or more?

In reality, many factors influence whether one large or several small reserves are more effective for achieving conservation goals (Soul and Simberloff 1986), so a more useful question for conservation decision making concerns the circumstances in which each approach is favoured (Williams et al. 2005; Tjørve 2010). If the aim is to protect multiple species, both approaches can enhance species richness as described above, with the expectation that the lower the proportional overlap in species among sites, the more effective is a multi-reserve approach (Connor and McCoy 2001; Tjørve 2010). However, the dynamic and transient responses of species distributions to rapid environmental change add some new provisos to this general guideline. For example, a greater number of species are expected to suffer delayed extinctions following habitat loss in landscapes with smaller rather than larger reserves (Kuussaari et al. 2009) and under climate change, one must also factor in the location of these sites and whether they remain climatically suitable for their focal species (Hodgson et al. 2009a, b). Where sites are forecast to remain climatically suitable, large reserves will benefit species with poor dispersal capability (Hodgson et al. 2009a, b). Conversely, for species with high dispersal rates, it is recommended to focus on patch number initially before increasing area (Nicol and Possingham 2010), enabling species to utilise the “stepping-stones” and shift in response to warming temperatures (Hodgson et al. 2012).

In the context of increasing extreme weather events, the distinction between the benefit of large reserves for habitat-interior species and small patches for edge species is exacerbated (Bender et al. 1998). Edge species are often more vulnerable to climate variability, especially when confined to small fragments of remaining habitat exposed to extreme weather events (e.g. Powell and Wehnelt 2003). Though larger reserves can be viewed as disadvantageous for species residing in ecotones or edge habitats (Bender et al. 1998), this is only a limitation in reserves consisting mainly of homogeneous habitat. Larger sites do tend to offer high levels of heterogeneity (Connor and McCoy 2001), accommodating pockets of habitat which can create the desired “edges” for these species within the reserve itself.

For species such as the many amphibians that are vulnerable to increasingly common disease epidemics amid a warming climate (Harvell et al. 2002; Pounds et al. 2006), more, smaller sites could provide local refuges from disease. Similarly, more sites are effective for species susceptible to environmental catastrophes as the risk of extinction is spread over several locations (Groeneveld 2005) and increases the chance of recolonization from nearby sites (Schnell et al. 2013). Nevertheless, threshold effects could render smaller sites too small to act as sources for range shifts, especially for those species with highly fragmented distributions or narrow geographic ranges (Pimm et al. 2014). When reserves are too small, wide-ranging species such as carnivores can leave the sites, heightening both human-wildlife conflict and carnivore mortality (Woodroffe and Ginsberg 1998). Species which congregate in relatively small areas at varying stages of their lifecycle (e.g. see BirdLife International 2008), however, could benefit from the presence of several smaller reserves provided they are situated in locations corresponding to resources favouring aggregation. The importance of the spatial context and surroundings of sites also appear to be more important than site area for exposure to invasive species, since sites surrounded by protected landscapes can have fewer invasive species than those amongst areas with varying land-uses (Pyšek et al. 2002).

Much attention surrounding the SLOSS debate has focused on the biological benefits of each strategy (see *Space for Nature* above). But in cases where there are no clear biological grounds on which method is likely to be best, how should we determine what is most practical? The economic aspects associated with the contrasting methods were conventionally not considered by theory (Groeneveld 2005), yet adopting cost-effective approaches is fundamental to meet ambitious biodiversity targets with limited funding (McCarthy et al. 2012) whether working on a fixed budget to capture as much biodiversity as possible (maximum coverage), or aiming to conserve a set amount of biodiversity for the minimum cost (minimum set) (Albuquerque and Beier 2015). Creating large sites could be more economical in terms of creation and management (Williams et al. 2005) as they start to rely on natural processes (Lawton et al. 2010) compared to managing smaller, individual sites. Overexploitation of species and habitats is a continuing challenge for biodiversity (Millennium Ecosystem Assessment 2005), thus the costs and feasibility of reserve protection against these threats will inevitably affect decisions. While the costs of internal monitoring (e.g. through transect surveys) of large sites versus small sites of equivalent area are comparable, notably less external surveillance is required for fewer, large sites with lower perimeter lengths (Ayres et al. 1991) and may be less at risk from poaching events (Di Minin et al. 2013). Enhancing the provision of ecosystem services promotes the ability of the environment to enhance human health and well-being, and lowers exposure to anthropogenic disturbances (Mitchell et al. 2015). But despite the expectation of greater diversity in large sites, whether large sites can enhance ecosystem function and the delivery of these services, relative to multiple smaller sites, remains equivocal. Nonetheless, with continuing land-use change leading to an increasingly fragmented landscape, there are frequently situations where it is physically not possible to create large sites due to surrounding land ownership or social and/or cultural costs of using a particular space (Williams et al. 2005). Moreover, people are altering their behaviour in response to climate change (Chapman et al. 2014), shifting agricultural regimes, modifying transport routes and building coastal defences, for example (see Segan et al. 2015). These indirect impacts of climate change can create additional barriers to creating large sites for conservation. In such cases, setting aside more, smaller sites for wildlife or opting for another strategy altogether, is often the only option.

## Exploiting connectivity

Site isolation plays a fundamental role in the theories of island biogeography and metapopulation biology by determining colonization rates (MacArthur and Wilson 1967) (Moilanen and Hanski 1998) and the Rescue Effect (Brown and Kodric-brown 1977). As human land conversion has greatly increased habitat isolation (Bennett 2003; Nicol and Possingham 2010), connectivity is often promoted to counteract biodiversity loss associated with habitat degradation (Williams et al. 2005; Donald and Evans 2006; Lees and Peres 2008). Connectivity is now also fundamental to facilitate species range shifts in response to climatic change (Lawson et al. 2012; Thomas et al. 2012; Lawson et al. 2013) and is thus commonly recommended for climate change adaptation (Heller and Zavaleta 2009).

But in today’s landscapes, increasing impacts from invasive species, pollution, disease and extreme weather events (Millennium Ecosystem Assessment 2005) present possible counterarguments for enhancing connectivity, given evidence that greater connectivity can lead to more rapid spread of catastrophic events (e.g. Laine 2004) and invasive species (Simberloff and Cox 1987; Dover and Settele 2009). Recent research has demonstrated that the deformed wing virus epidemic in the European honeybee *Apis mellifera*, is driven by movement of pollinator populations and spread of the mite *Varroa destructor,* and greater functional connectivity (i.e. the behavioural response of an organism to landscape features [Tischendorf and Fahrig 2000]) for the vectors of the disease therefore enhance its potential to spread to other wild pollinators (Wilfert et al. 2016). As a result, large distances between sites and regulated movement are now necessary to reduce the spread of disease, invasive species, predators, and the impacts of environmental events such as fire or hurricanes (Williams et al. 2005). Networks of sites that are well connected in terms of the dispersal capabilities of target species, but remain fragmented with respect to the transmission of disease (Huxel and Hastings 1999; Hartley and Kunin 2003; Williams et al. 2005) or susceptibility to regionally correlated environmental variation, would represent win–win situations, although providing the information required to define this optimal level of aggregation is challenging (Williams et al. 2005).

Connectivity has traditionally focused on habitat corridors, which can include natural or man-made linear features such as rivers, canals, hedgerows and railway embankments (Lawton et al. 2010). Managing the matrix between sites is often advocated as a means of making the space between pockets of protected areas amid intense land use more permeable to allow for species movement (Lees and Peres 2009). In addition, increasing the number of sites and aggregating them within the dispersal distance of focal species enhances movement, though could reduce opportunities for range expansion if not adequately spaced (Magris et al. 2014). More recently, research has begun to highlight the role of the other strategies associated with reserve design for enhancing connectivity. Local population dynamics in addition to distance between patches are essential for determining functional connectivity (i.e. potential rates of immigration). Habitat area and quality increase the size and stability of source populations for dispersal and hence rates of immigration to other patches (Hodgson et al. 2009c). Recent research has shown that stable abundance trends are more important than dispersal ability in influencing rates of range expansion in British butterflies (Mair et al. 2014), and reproductive rates of wetland vertebrates had more influence on immigration rates than species mobility (Quesnelle et al. 2014). Thus promoting population growth through maintaining habitat quality and size is essential, and directing efforts exclusively to structural connectivity (focusing on the physical structure of the landscape [Tischendorf and Fahrig 2000]) is only beneficial under specific circumstances.

The primary purpose of enhanced connectivity (both functional and structural) is to augment species movement between sites, which is becoming increasingly more important across landscapes as range shifts are forced by climate change. Therefore, the value of increased structural connectivity alone depends on whether persistence or range expansion are limited by the dispersal ability of species relative to the existing configuration of habitats (Moilanen and Hanski 1998). The most dispersive species may not benefit from increased connectivity (Bennett 2003), but highly sedentary species may only benefit if connectivity is increased within the dispersal range of the species concerned (e.g. Doerr et al. 2011; Johst et al. 2011). With ongoing fragmentation, distances between habitats can exceed dispersal capacity for many species (Dennis et al. 2013). As a result, guidelines to identify the level of isolation of sites relative to species dispersal capacity at which enhanced connectivity most benefits regional persistence, would help to increase the effectiveness of landscape-scale conservation (Lees and Peres 2009). Such approaches could benefit the species with intermediate dispersal capabilities that have declined more than either the most sedentary or mobile species (Thomas 2000). When considering the level and capability of dispersal, it is also necessary to consider how dispersal mode differs within and between taxa (Hodgson et al. 2011a). Animal-dispersed plants, for example, can increase following the introduction of corridors for animals, whereas wind-borne dispersers may be unaffected (Brudvig et al. 2009).

Finding the space to make sites bigger across the modern human-dominated landscape is becoming increasingly problematic. As a result, enhancing connectivity may be essential for species requiring access to the resources needed (Benton et al. 2003), especially those with varying needs at various stages of their lifecycles (Fahrig 2003) or with seasonal food requirements (Donald and Evans 2006), and may also encourage animals to reside within appropriate habitats, reducing human-wildlife conflict (Hartter and Southworth 2009). Establishing corridors between sites can be expensive (Dennis et al. 2013), in which case utilising man-made structures or existing natural connections is a plausible solution. Managing matrix habitat may be needed when a location offers a fragmented network of protected areas surrounded by intense land use. In return, this not only provides species with an increased capacity to shift, it enhances the ability of the environment to provide a range of ecosystem services such as pollination, human well-being and air quality. Nevertheless, in areas vulnerable to spatially autocorrelated contagion-like extinction pressures (Channell and Lomolino 2000), connectivity should be avoided; instead, opting for widely separated reserves will be more effective (Hartley and Kunin 2003).

## Interplay between approaches

In reality, it is clearly not a straightforward case of selecting one approach; opting for a particular strategy can impact the ability to achieve, or even the requirement for another. Previous work has focused on the effect of habitat quality and area in enhancing functional connectivity between sites (e.g. Hodgson et al. 2009c, 2011a but see also Doerr et al. 2011), thus choosing to develop quality or area can be an effective option for improving connectivity if required. Authors have also alluded to the fact that focusing on quality can mean there is less need to create new areas for wildlife (Lawton et al. 2010), though improving connectivity directly will ensure that species can actually reach these high quality habitats (Root 1998). In any case, enlarging sites reduces the need for connectivity (Rosenberg et al. 1997; Haddad 1999; Dennis et al. 2013) as these areas start to act as stand-alone reserves (Williams et al. 2005), providing they reside in climatically suitable or stable areas, and also tend to offer the benefits of habitat heterogeneity when areas are sufficiently large to host a broad range of habitats. Likewise, the creation of corridors can effectively increase the size of the site (Benton et al. 2003; Noel et al. 2006; Lawson et al. 2013) and so remains a useful alternative when the creation of big sites is not an option. But where the designated area of land for conservation purposes is limited in size within conservation planning, the creation of corridors could mean that the area of the sites themselves would have to be smaller to meet the overall area on offer (Rosenberg et al. 1997). Should more, smaller sites prove to be the best option, these areas can themselves act as stepping stones, promoting connectivity (Hodgson et al. 2009c) and simultaneously offering habitat heterogeneity (Dover and Settele 2009). Although if these sites are separated to protect against climatic disturbance, this could negatively affect the ability to suitably enhance connectivity and facilitate range shift if required (Magris et al. 2014).

## Moving forward

Although context-dependent, formulating a series of generic rules would provide a much needed starting point to assist conservation practitioners involved in decision-making regarding the planning and management of protected areas amid future threats. Given the current and future constraints imposed on biodiversity and the acute shortage of funding for effective conservation, it is not always possible to implement the creation of bigger, better and more joined sites for conservation and difficult choices between these strategies will often need to be made. With increasing land-use change, for example, creating bigger sites is rarely possible within fragmented landscapes, whilst restoring increasingly degraded habitat through in situ management can be expensive and time consuming (Possingham et al. 2015). Responding to an increase in invasive species, pollution and disease requires protection and management to be undertaken in widely spaced locations, bearing in mind the trajectories of climate change and routes species may follow as they shift their distributions in response (Loarie et al. 2009; Early and Sax 2011). It is now widely accepted that conservation strategies should account for climate change (Jones et al. 2016) and the inevitable need to adapt to changing temperatures, cope with environmental extremes and shift in response to climatic changes. In this case, focusing on habitat quality is the most effective strategy (Greenwood et al. 2016) but specifically how to approach this depends on a series of factors. Bigger sites and multiple smaller sites each offer benefits for climate change adaptation, whilst the functional connectivity required for this challenge can be improved through a focus on other strategies which encourage stable abundance. Indirect impacts associated with climate change have seldom been recognised in the literature (Chapman et al. 2014) but can further complicate the ability to adopt particular strategies, or the overall effectiveness of those employed. With the potential of people to shift agricultural practices, for example, utilising numerous smaller sites may enable people to exploit areas of land in between, as opposed to entering those areas designated for wildlife (Bradley et al. 2012).

The literature associated with conservation planning has vastly progressed since the origin of reserve design theory presented by Diamond (1975). Many of the ideas proposed by classical theory still apply in the context of modern-day pressures, such as the ability of larger sites to deal with stochasticity as a result of high carrying capacities, and enhance the colonization of surrounding habitat from large source populations. Other recommendations become even more important when we factor in rapid environmental change, such as the provision of source populations provided by optimal habitat for species’ range shifts, the buffering effect supplied by large populations within larger sites, and the reduced extinction risk of multiple smaller sites from correlated environmental events. Meanwhile, there are evidently cases where ideas from conventional theory no longer apply. Single large sites are prone to extinction from increasing environmental disturbances, counteracting the traditional desire to maintain structural connectivity between sites, alongside the fact that it is simply not possible in today’s landscapes to create single large sites for nature, where levels of biodiversity may be high and often coincide with high human populations.

### Decision-making framework

In essence, the most effective strategy in the context of twenty-first century pressures depends on circumstance, but by considering the goals of conservation and the characteristics of biota for which conservation is needed, it is possible to make informed choices about which strategy is likely to be best (Table 1). Nevertheless, practical considerations such as financial costs, reserve protection (day to day and in the future) and site monitoring are also important and are seldom considered in studies of reserve design (Groeneveld 2005). From the resulting recommendations shown in Table 1, size and connectivity represent the most prominent strategies amongst the considerations highlighted. However, it is noteworthy that this may not consistently be the case, particularly when focusing on issues associated with modern-day conservation including economic constraints, extent of habitat fragmentation, vulnerability to climate change and risk of disease and environmental disturbance. Upon adopting a particular conservation strategy, there are evidently multiple valid options for a particular situation (Table 1). Our review of the literature suggests that, amid twenty-first century challenges, habitat quality and area should be the priority (as in Hodgson et al. 2009c; Lawton et al. 2010); enhancing, amongst other things, the ability of species to shift in a changing climate, cope with environmental extremes and promote species richness and population viability. This offers the additional advantage of being more cost-effective than focusing on connectivity between sites, especially when protecting currently intact habitat. The exception to this rule is within existing fragmented landscapes, where area and connectivity become more important than quality (Moilanen and Hanski 1998). Since enhancing the quality and/or quantity of sites offer many of the benefits associated with connectivity, encouraging connectivity alone is only supported in a few circumstances. Despite this, more connectivity is generally considered better than isolation, aside from populations exposed to spatially contagious threats such as disease epidemics, but at low risk from climate change and hence not expected to require the ability to shift their range at least over the short term.

The principles of this framework can effectively be used to provide solutions to twenty-first century issues (Box 1) where conservation continues to struggle to find answers to complex debates; highlighting the role of scientific theory in modern day conservation planning.

**Box 1:** Decision making in the real world: a case study of land spare versus land share

Alongside threats from habitat change, climate change and invasive species, one of the greatest threats to global biodiversity is the need to balance the increasing demand for food security with conservation (Green et al. 2005; Donald and Evans 2006; Fischer et al. 2008; Edwards et al. 2010; Balmford et al. 2012). Land sparing involves the preservation of natural areas for wildlife, segregated from a smaller area of land for intensive agriculture, while land sharing, or wildlife-friendly farming, involves the spatial co-occurrence of agriculture and conservation (Phalan et al. 2011; Tscharntke et al. 2012; Grau et al. 2013). Land sharing has been encouraged, particularly in Europe, with the support of agri-environment payments through the Common Agricultural Policy and various other certification schemes worldwide. These include the Conservation Reserve Program in the USA (Green et al. 2005; Kleijn et al. 2011; Hulme et al. 2013) and the Australian Landcare Program (Kleijn et al. 2011); aiming to cover the net losses that occur from avoiding more intensive farming methods (Lawton et al. 2010), and provide support to those farmers who opt to make environmental improvements to their land (Donald and Evans 2006)
The land share, land spare debate epitomises the difficult choices faced in landscape-scale conservation planning: on one hand, a high quality (relatively homogenous) but smaller area of spared land for wildlife; on the other, lower quality but larger areas of heterogeneous habitat shared with farming (Green et al. 2005; Fischer et al. 2008; Adams 2012; Balmford et al. 2012). As with the trade-offs associated with reserve design, both approaches have strengths and weaknesses (Edwards et al. 2010). Land sharing can enhance and restore connectivity by creating softer barriers to dispersal between areas of more natural habitat (Donald and Evans 2006; Fischer et al. 2008; Heller and Zavaleta 2009; Dover and Settele 2009). Sharing also encourages the creation of new wildlife sites (Donald and Evans 2006; Dover and Settele 2009; Lawton et al. 2010) although more land, potentially previously intact, must be cultivated to balance the fact that overall yield is low (Green et al. 2005; Balmford et al. 2012; Hulme et al. 2013; Chandler et al. 2013). Nevertheless, this may mean that more land is protected in some way (Balmford et al. 2012). In contrast, land sparing can boost species populations (e.g. Phalan et al. 2011), particularly those of greatest conservation concern (Hulme et al. 2013), and thus assist with climate change adaptation through abundant source populations. It can also increase overall species richness (Edwards et al. 2010; Chandler et al. 2013) due to more native habitat (Hulme et al. 2013) and because many wild species cannot survive in even the most wildlife friendly farmland (Tscharntke et al. 2012). However, some species are specifically adapted to agricultural landscapes (Benton et al. 2003), particularly in landscapes with a long-history of disturbance (Donaldson et al. 2016). Land sparing usually produces higher yields (Grau et al. 2013), potentially reducing deforestation rates since there is less pressure to log other areas to meet demand (see Green et al. 2005) and more recently reported to save on greenhouse gas emissions as a result of less land conversion to meet demand for agriculture (Balmford et al. 2012)
Amongst the confounding benefits discussed extensively in the literature, our decision making framework can be used to demonstrate how theory associated with reserve design can help provide solution to this intensive debate (Table 2). The homogeneous quality associated with spared land provides benefits to specialist species, boosts populations of species vulnerable to climate warming, and provides smaller sites suitable for stationary animals with small range sizes. Providing more, smaller sites can also enhance the capacity for range shift across the landscape in response to climatic change. Meanwhile, land sharing generally enhances connectivity between sites, offering benefits to migratory species and those with low dispersal capabilities and/or large range sizes, but equally may spread the risk of extinction from correlated weather events and disease. Providing the landscape remains relatively fragmented with respect to these risks, the heterogeneity associated with land sharing can help buffer the effects of variable environmental disturbances. Land sharing is also an appealing option in areas where wildlife and low intensity forms of agriculture have coexisted for long periods of time, such as parts of Europe (Fischer et al. 2008; Hodgson et al. 2010), where species are tolerant to disturbance from such activities (Grau et al. 2013). Conversely, in areas with high potential agricultural activity that do not coincide with those of high biodiversity value, it is possible to zonate land and opt for a land sparing approach (Hodgson et al. 2010). However, with environmental change, crop suitability may also shift (Bradley et al. 2012) leading people to encroach on spared land. In this sense, suitable areas for people to farm with climate change could be equally as important as providing suitable areas for species’ ranges to shift, or alternatively opt for a land sharing approach where both have the potential to move. Finally, this challenge highlights the importance of practical considerations (Table 2), with site ownership, planning and governance being amongst the most fundamental factors leading to the most appropriate option available

**Table 2:** The prevalent factors derived from a range of scientific studies associated with the theory of reserve design influencing solutions to the land spare, land share debate

Factor			Land spare^a^	Land share^b^	Justification	Reference(s)
Species traits	Habitat preference	Specialist	✓		Land sparing provides higher quality, natural habitat suitable for specialists, whilst generalists can exist in lower quality habitats	Green et al. (2005), Devictor et al. (2008), Fischer et al. (2008), Hulme et al. (2013), Ye et al. (2013)
		Generalist		✓		
	Habitat requirements	Migratory		✓	Some species require a variety of habitats (heterogeneity), continuity (connectivity) and/or large areas to complete life cycle	Donald and Evans (2006), Johnson (2007), Fischer et al. (2008)
		Stationary	✓			
	Human disturbance	Sensitive	✓		Land sparing involves less disturbance to wildlife since area is spared for them	Green et al. (2005), Grau et al. (2013)
		Tolerant		✓		
	Range size	Small	✓		Land sparing involves a smaller area of high quality land designated for wildlife, while land sharing settles for a lower quality but much larger area of land for wildlife	Phalan et al. (2011), Hulme et al. (2013)
		Large		✓		
	Dispersal capability	High	✓		Land sharing enhances connectivity through soft barriers to dispersal between areas of natural habitat	Donald and Evans (2006), Fischer et al. (2008), Heller and Zavaleta (2009), Dover and Settele (2009)
		Low		✓		
	Population viability	High		✓	Land sparing can boost species populations	e.g. Phalan et al. (2011)
		Low	✓			
Threats	Climatic change	High vulnerability, low variability	✓		Higher quality spared land can provide source populations for climate adaptation and assist with capacity for range shift	Phalan et al. (2011)
		High variability, low vulnerability		✓	Sharing is associated with a heterogeneous landscape, thus buffers environmental disturbances (providing landscape remains relatively fragmented to spread extinction risk)	Fischer et al. (2008)
Practical	Ownership	Multiple		✓	Land sparing is not possible with multiple owners	Adams (2012)
		Single	✓			
	Planning	Strong	✓		Land sparing requires a strong and effective planning approach to be successful and not detrimental to wildlife.	Adams (2012)
		Weak		✓		
	Governance	Strong	✓		Land sparing is difficult to implement in countries with weak governance, requires strict policy mechanisms to be effective and ensure areas farmed are restricted	Edwards et al. (2010), Hodgson et al. (2010), Adams (2012)
		Weak		✓		

^a^ Typically offers homogeneous, smaller, less connected sites ^b^ Generally composed of heterogeneous, larger, more connected habitat

## Conclusion

As threats to biodiversity and competing demands for land increase, the effective targeting of conservation resources is increasingly urgent. While many authors have concluded that simple concrete rules for reserve design do not exist, the knowledge base is extensive. The very broadness and complexity of the literature regarding reserve design has come to represent a challenge to those adopting measures to promote landscape-scale conservation, and new threats to biodiversity conservation demand a reevaluation of classical ideas for reserve design. We have synthesised and explored existing knowledge to provide updated, generic guidance to decision makers engaged in landscape-scale conservation planning and practice in the context of levels of environmental change and biotic consequences that were not envisaged only decades ago. Ambitious global biodiversity targets are set and funding for conservation is notoriously limited. By providing an evidence-based framework that summarises the circumstances under which each strategy is best, we hope to provide increased clarity to inform urgent, cost effective modern-day conservation decision-making.
